# Evidence-Based Assessment of Pesticide-Related Nephrotoxicity: Clinical Outcomes, Experimental Data, and Molecular Signatures

**DOI:** 10.3390/ijms27093970

**Published:** 2026-04-29

**Authors:** Hsin-Yi Lu, Yung Chang, Chih-Kang Chiang

**Affiliations:** 1 Applied Toxicology Division, Agricultural Chemicals Research Institute, Ministry of Agriculture, Taichung 413001, Taiwan; d10447005@ntu.edu.tw; 2 Graduate Institute of Toxicology, College of Medicine, National Taiwan University, Taipei 100233, Taiwan; d06447001@ntu.edu.tw; 3 Department of Integrated Diagnostics & Therapeutics, National Taiwan University Hospital, Taipei 100225, Taiwan

**Keywords:** pesticide nephrotoxicity, acute kidney injury, chronic kidney disease, proximal tubular injury, glyphosate, paraquat, organophosphate insecticides, renal biomarkers

## Abstract

Pesticide exposure is a plausible but incompletely characterized contributor to kidney injury. This review integrates current clinical, epidemiologic, experimental, and mechanistic evidence on pesticide-related nephrotoxicity, focusing on glyphosate-based herbicides, paraquat, organophosphate insecticides, and atrazine. A structured search of PubMed and Web of Science identified English-language studies published between January 2015 and February 2026. Of 635 records screened, 61 human studies were retained for full-text evaluation, and relevant animal, in vitro, and regulatory sources were additionally reviewed for mechanistic interpretation. Across pesticide classes, the proximal tubule emerged as the most consistent renal target, although downstream pathways differed, including oxidative stress, mitochondrial dysfunction, transporter disruption, endoplasmic reticulum stress, inflammation, apoptosis, ferroptotic signaling, and fibrotic remodeling. Human evidence was strongest for acute kidney injury following severe poisoning, whereas associations between chronic occupational or environmental exposure and chronic kidney disease or end-stage renal disease were more limited and heterogeneous. Biomarkers including kidney injury molecule-1 (KIM-1), neutrophil gelatinase-associated lipocalin (NGAL), β_2_-microglobulin, cystatin C, interleukin-18 (IL-18), cytochrome c, and 8-hydroxy-2′-deoxyguanosine (8-OHdG) often detected early tubular stress before abnormalities appeared in conventional renal indices. Overall, pesticide nephrotoxicity is best conceptualized as a spectrum of mechanism-specific tubular injury signatures, supporting a shift toward biomarker-informed early detection, improved hazard identification, and more mechanistically grounded risk assessment.

## 1. Introduction

Pesticides remain indispensable to modern agriculture because they protect crop yield, crop quality, and post-harvest storage, thereby supporting global food production. However, their extensive and intentional release into the environment also creates broad opportunities for human and ecological exposure. Global agricultural pesticide use has increased substantially in recent decades, raising concerns about contamination of soil, water, and air, as well as adverse effects on non-target organisms, biodiversity, and human health [1,2]. Human exposure occurs through multiple routes, including occupational handling, residential use, spray drift, and ingestion of residues in food and drinking water, with agricultural workers representing one of the most directly exposed populations [1,3]. In parallel, kidney disease, particularly acute kidney injury (AKI) and chronic kidney disease (CKD), has become a major global health burden, with CKD alone affecting hundreds of millions of people worldwide and contributing substantially to morbidity, mortality, and health-care costs [4]. This concern is especially relevant because the kidney is inherently vulnerable to xenobiotic injury: its high blood flow, the tubular concentration of filtered chemicals, and the energy-dependent transport processes of proximal tubular cells can all increase intracellular toxicant burden [5,6]. Together, these considerations support the plausibility that pesticide exposure contributes to renal injury, yet the available evidence remains fragmented across poisoning studies, epidemiologic investigations, animal models, and mechanistic research. Accordingly, this review is organized at either the pesticide-class or individual-compound level, depending on the structure of the available evidence and the most informative unit for mechanistic interpretation: glyphosate-based herbicides and organophosphate insecticides are considered as grouped exposures because the literature commonly supports formulation- or class-level synthesis, whereas paraquat and atrazine are examined individually because each has a distinct toxicological profile and a sufficiently developed kidney-related evidence base to justify compound-specific evaluation. This review therefore provides an evidence-based assessment of pesticide-related nephrotoxicity by integrating clinical outcomes, experimental findings, and molecular signatures, with emphasis on glyphosate-based herbicides, paraquat, organophosphate insecticides, and atrazine.

## 2. Methods

### 2.1. Literature Search Strategy

A structured literature search was conducted using PubMed and Web of Science to identify studies evaluating associations between pesticide exposure and kidney-related outcomes. PubMed queries incorporated both controlled vocabulary (Medical Subject Headings, MeSH) and free-text terms, whereas Web of Science searches employed topic-based free-text queries.

Search terms combined pesticide-related exposure descriptors (e.g., pesticides, insecticides, herbicides, fungicides, organophosphates, carbamates, pyrethroids, glyphosate, paraquat, atrazine) with renal outcome terms (e.g., kidney disease, acute kidney injury [AKI], chronic kidney disease [CKD], end-stage renal disease [ESRD], renal dysfunction, nephrotoxicity).

The search was restricted to English-language publications published between January 2015 and February 2026 to capture recent epidemiological and mechanistic developments. In addition, reference lists of relevant articles were manually screened to identify further eligible studies.

### 2.2. Study Selection and Eligibility Criteria

Eligible human studies included prospective cohort, case–control, and cross-sectional epidemiological studies, as well as clinical investigations and case series reporting renal outcomes associated with pesticide exposure. Studies were excluded if they lacked primary data, did not clearly define renal outcomes, involved kidney injury primarily attributable to major non-pesticide exposures, or provided insufficient assessment of pesticide exposure.

After application of the predefined date and language restrictions, 635 records were identified through searches of PubMed and Web of Science, with an additional 15 records identified through manual screening of reference lists. In total, 650 titles and abstracts were screened for relevance to pesticide exposure and kidney-related outcomes. A total of 589 records were excluded based on the predefined eligibility criteria. The remaining 61 articles underwent full-text review, of which 28 were excluded, leaving 33 human studies for inclusion in the scoping review.

**Figure 1:**
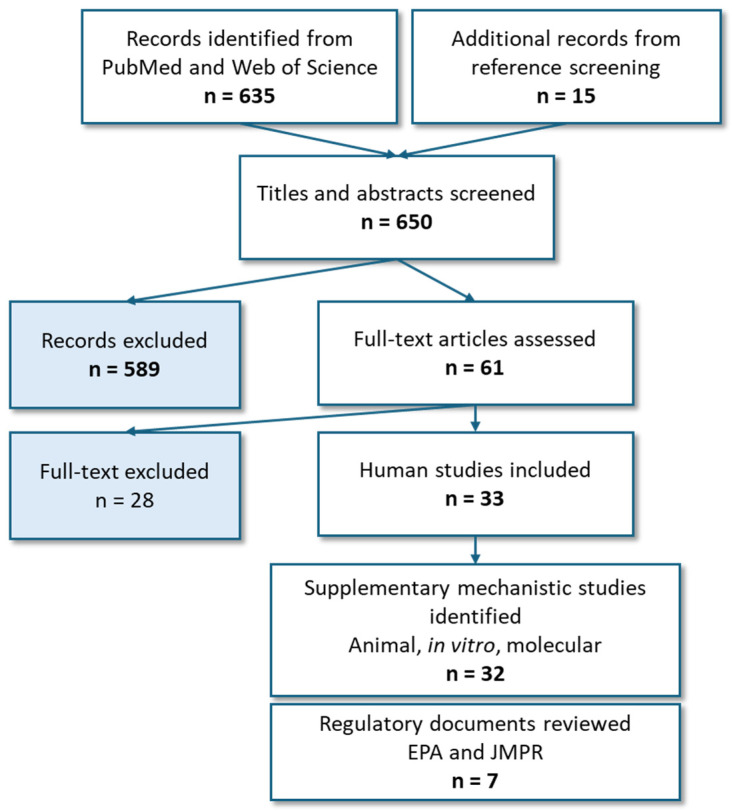
Flow chart of study identification, screening, eligibility assessment, and evidence mapping for the scoping review of pesticide exposure and kidney-related outcomes.

For pesticides with at least limited human evidence of nephrotoxicity, supplementary targeted searches were conducted to identify relevant mechanistic evidence, yielding 32 additional studies spanning animal, in vitro, and molecular research. In addition, 7 publicly accessible regulatory documents from the U.S. Environmental Protection Agency (EPA) and the Joint FAO/WHO Meeting on Pesticide Residues (JMPR) were reviewed to extract relevant toxicological endpoints and target organ information (Figure 1).

### 2.3. Outcome Definitions

Kidney-related outcomes were interpreted using established international clinical criteria to ensure consistency across studies.

#### 2.3.1. Acute Kidney Injury (AKI)

Acute kidney injury (AKI) was defined according to established clinical criteria, primarily the Kidney Disease: Improving Global Outcomes (KDIGO) Clinical Practice Guideline for Acute Kidney Injury, with earlier studies applying the Acute Kidney Injury Network (AKIN) criteria or the RIFLE classification (Risk, Injury, Failure, Loss, and End-stage kidney disease). These definitions represent sequentially harmonized frameworks for AKI diagnosis [7,8,9].

#### 2.3.2. Chronic Kidney Disease (CKD)

Chronic kidney disease (CKD) was defined according to KDIGO guidelines as abnormalities of kidney structure or function persisting for more than three months, including a sustained reduction in estimated glomerular filtration rate (eGFR < 60 mL/min/1.73 m^2^) or other markers of kidney damage [10].

Caution was applied when interpreting cross-sectional studies that classified CKD based on a single eGFR measurement, as persistence over time is required for formal diagnosis.

#### 2.3.3. Chronic Kidney Disease of Unknown Etiology (CKDu)

Chronic kidney disease of unknown etiology (CKDu) was defined according to criteria established by the Sri Lanka Ministry of Health. CKDu was diagnosed among individuals with CKD in the absence of known traditional causes, including diabetes mellitus, chronic or severe hypertension, snakebite, urological disease of known etiology, or glomerulonephritis. Additional criteria included normal glycosylated hemoglobin levels (HbA1c < 6.5%) and controlled blood pressure (<160/100 mmHg untreated or <140/90 mmHg on up to two antihypertensive agents) [11].

It should be noted that CKDu lacks a universally standardized definition. While Sri Lankan criteria are commonly applied in endemic regions, other geographic settings (e.g., Mesoamerica) use modified exclusion-based definitions. This heterogeneity was considered when evaluating the strength of evidence linking pesticide exposure to CKDu.

#### 2.3.4. End-Stage Renal Disease (ESRD)/End-Stage Kidney Disease (ESKD)

End-stage renal disease (ESRD), increasingly referred to as end-stage kidney disease (ESKD), was defined according to the United States Renal Data System (USRDS) as irreversible kidney failure requiring renal replacement therapy (RRT), including chronic dialysis or kidney transplantation [12].

### 2.4. Evidence Grading Approach

An outcome-specific qualitative framework was applied to classify the strength of human evidence linking individual pesticides to kidney-related outcomes. This approach was informed by established weight-of-evidence principles in regulatory toxicology and environmental health, including frameworks developed by the World Health Organization/International Programme on Chemical Safety (WHO/IPCS), the U.S. Environmental Protection Agency (EPA), and systematic review methodologies such as the Navigation Guide [13,14,15].

Evidence grading was based on the overall balance of six considerations: study design, consistency across studies, exposure assessment quality, presence or absence of an exposure–response relationship, clinical relevance of the renal endpoint, and vulnerability to bias or confounding. Biological plausibility derived from experimental and mechanistic studies was considered supportive but not determinative for causal inference. Grading was conducted separately for acute kidney injury (AKI) and chronic kidney disease (CKD/ESRD) to distinguish high-dose acute toxic syndromes from chronic progressive renal injury.

Evidence strength was categorized as follows:

**Strong evidence**

- Multiple high-quality studies with consistent findings across independent populations or settings;
- At least one well-designed prospective cohort or clinical poisoning cohort with clinically robust renal endpoints;
- Clear exposure–response relationship or other strong internal gradient supporting inference.

**Moderate evidence**

- At least one well-designed cohort study or multiple generally consistent observational studies;
- Clinically relevant renal outcomes and/or coherent biomarker evidence of renal injury or dysfunction;
- Exposure–response relationship is present but limited, indirect, or not consistently demonstrated across studies.

**Limited evidence**

- Suggestive but inconsistent findings, usually based mainly on cross-sectional or case–control studies;
- Supportive clinical or biomarker signals, but important limitations in exposure assessment, outcome ascertainment, sample size, or control of confounding;
- Case reports or case series may contribute supportive evidence, particularly for acute poisoning, but have limited generalizability.

**Weak or insufficient evidence**

- Sparse, isolated, or methodologically weak data;
- Evidence limited to single case reports, very small uncontrolled series, or studies with major design limitations;
- No reproducible pattern or meaningful evidence of exposure–response relationship.

In borderline cases, judgments were based on the combined balance of the six considerations rather than any single criterion in isolation. Thus, “limited to moderate” was used when the evidence indicated a recurrent and biologically coherent signal but remained constrained by important limitations, whereas “moderate to strong” was used when the evidence approached the features of strong evidence but fell short of fully meeting all criteria, such as consistency, number of high-quality studies, or exposure–response support.

For clarity of presentation, evidence was summarized at two complementary levels. First, pesticide-specific summary tables are provided at the end of each pesticide section to integrate the principal human study context, dominant kidney outcomes, key biomarkers or supportive findings, pathological interpretation, and overall strength of evidence. Second, a later cross-pesticide synthesis table summarizes the principal molecular signatures shared across pesticide classes, while experimental models and study descriptors supporting mechanistic interpretation across glyphosate-based herbicides, paraquat, organophosphates, and atrazine are summarized in Supplementary Table S5.

## 3. Results/Evidence Synthesis

### 3.1. Glyphosate and Glyphosate-Based Herbicides

#### 3.1.1. Clinical Outcomes

##### Exposure Context and Relevance

Glyphosate is a broad-spectrum, non-selective herbicide belonging to the organophosphorus class of compounds, specifically a phosphonate, and is widely used in agriculture, forestry, and residential weed control. Unlike organophosphate insecticides, glyphosate does not inhibit acetylcholinesterase, reflecting a distinct toxicological profile. Its extensive use results in diverse opportunities for human exposure, primarily through occupational handling by pesticide applicators and agricultural workers, as well as through dietary intake and environmental residues in water and soil [16,17].

Toxicokinetic studies indicate that glyphosate is only partially absorbed following oral exposure (approximately 30–40%), undergoes minimal metabolic transformation, and is eliminated largely unchanged in urine and feces within 24–48 h, suggesting limited bioaccumulation potential. The primary metabolite, aminomethylphosphonic acid (AMPA), generally represents only a minor fraction of the absorbed dose [16,17].

##### Acute Kidney Injury in Poisoning Cohorts

Most human evidence linking glyphosate to kidney injury derives from studies of glyphosate-based herbicide (GBH) poisoning, in which toxicity reflects combined effects of glyphosate and formulation co-components, particularly surfactants (Supplementary Table S1). Acute ingestion can produce multi-organ toxicity, including metabolic acidosis, cardiovascular instability, and AKI [18,19].

Prospective biomarker studies provide mechanistic insight, showing early and significant elevations in structural kidney injury markers—including urinary interleukin-18 (IL-18), neutrophil gelatinase-associated lipocalin (NGAL), trefoil factor-3 (TFF3), cystatin C, and cytochrome c—in patients who subsequently develop AKI, with strong early diagnostic performance (AUC-ROC ≥ 0.8) [20]. This profile is consistent with predominant proximal tubular injury involving mitochondrial dysfunction and apoptotic signaling [21,22]. Elevated urinary cytochrome c supports a central role for mitochondrial injury, a finding reinforced by multi-toxicant cohort data indicating that mitochondrial damage may outweigh lipid peroxidation as a driver of toxic AKI [23].

Clinically, AKI occurs in a substantial proportion of severe poisonings and is closely associated with systemic toxicity. In a retrospective cohort, AKI incidence approached 45% and was linked to hypotension, electrocardiographic abnormalities, prolonged hospitalization, and increased mortality [24].

Importantly, formulation components appear to be major determinants of toxicity. The volume of ingested surfactant correlates more strongly with clinical complications—including AKI, hypotension, and respiratory failure—than glyphosate concentration alone, implicating co-formulants as key contributors [18].

Despite these findings, limitations remain, including the lack of direct glomerular filtration measurements, quantified systemic glyphosate exposure, and detailed characterization of surfactant composition. Overall, the evidence supports predominantly tubular, mitochondria-associated injury following high-dose GBH poisoning, although extrapolation to chronic low-level exposure remains uncertain.

##### Chronic Kidney Outcomes in Epidemiologic Studies

Evidence linking long-term glyphosate exposure to chronic kidney disease remains limited and inconsistent (Supplementary Table S1). In the Agricultural Health Study (AHS), a large prospective cohort of licensed pesticide applicators in the United States, no association was observed between glyphosate exposure and incident ESRD [12].

In contrast, several observational studies conducted in regions affected by chronic kidney disease of unknown etiology (CKDu) have reported associations between glyphosate exposure and impaired renal function. A case–control study in Sri Lanka found that occupational glyphosate use and consumption of well water contaminated with agrochemicals were associated with increased CKDu risk among farmers [11]. Cross-sectional biomonitoring studies in agricultural communities have similarly reported correlations between urinary glyphosate concentrations and renal injury biomarkers—including NGAL, β_2_-microglobulin, and albumin-creatinine ratio—together with modest reductions in estimated glomerular filtration rate [25].

Evidence in environmentally exposed populations is less consistent. A biomonitoring study of infants and young children detected urinary glyphosate in a subset of participants but found no association with kidney injury biomarkers such as KIM-1, NGAL, or albuminuria [26]. Conversely, a recent cross-sectional study in Mexican children reported that higher urinary glyphosate concentrations were associated with increased levels of the tubular injury biomarker KIM-1 despite normal glomerular filtration rates, suggesting the possibility of early subclinical tubular injury [27].

Overall, the available human evidence indicates moderate clinical evidence for AKI following acute glyphosate-based herbicide poisoning, whereas epidemiological evidence linking chronic environmental exposure to kidney disease remains limited and heterogeneous. The principal human evidence for glyphosate and GBHs is summarized in Table 1.

#### 3.1.2. Experimental and Molecular Mechanisms of Nephrotoxicity

Experimental evidence from rodent and renal tubular cell models indicates that glyphosate-associated renal injury primarily involves oxidative stress-mediated damage to renal tubular cells, with substantially greater toxicity observed for commercial formulations than for the pure active ingredient.

Subchronic studies in rats demonstrate renal dysfunction characterized by increased plasma creatinine and urea, reduced creatinine clearance, and proximal tubular degeneration and necrosis, accompanied by marked oxidative stress, including lipid and protein oxidation and depletion of antioxidant defenses such as superoxide dismutase, catalase, and glutathione [28]. These findings are supported by formulation-specific studies in adult male rats showing dose-dependent tubular necrosis, cortical distortion, glomerulosclerosis, and impaired renal filtration following exposure to glyphosate-based herbicides [29].

Comparative studies further indicate that formulation co-components substantially amplify nephrotoxicity. Exposure to commercial formulations increases serum urea, creatinine, and NGAL, enhances lipid peroxidation, suppresses membrane ATPases (Na^+^/K^+^-, Ca^2+^-, and Mg^2+^-ATPase), and is associated with greater renal glyphosate accumulation, whereas glyphosate alone produces minimal or adaptive responses at comparable doses [29]. Together, these findings implicate oxidative membrane injury and impaired tubular transport as key features of formulation-driven toxicity.

At the molecular level, one experimental study in HK-2 proximal tubular cells, supported by a parallel mouse model, suggests that glyphosate may induce proximal tubular apoptosis through N-methyl-D-aspartate receptor (NMDAR)-associated signaling, leading to Ca^2+^ influx, reactive oxygen species generation, and mitochondrial apoptotic responses (↑ Bax/Bcl-2, caspase-3 activation) [30]. In the same study, early increases in urinary β_2_-microglobulin and albumin, together with tubular cell exfoliation, supported impaired tubular reabsorption and epithelial injury. However, the involvement of NMDAR signaling has not been widely replicated and should be interpreted cautiously.

Developmentally extended exposure models in male Wistar rats indicate that prolonged low-dose exposure induces mild tubulointerstitial alterations without evidence of overt renal failure. Reported changes include increased *Havcr1* (*Kim-1*) expression, tubular swelling, interstitial inflammation, and altered transporter gene expression (e.g., *Slc14a1*, *Trpm6*, *Trpv5*), accompanied by modest or variable changes in conventional renal function markers [31].

Collectively, these findings support a mechanistic cascade in which glyphosate-based herbicides induce oxidative stress-driven mitochondrial dysfunction, leading to tubular epithelial injury, impaired reabsorptive capacity, and progressive tubulointerstitial damage. However, the relative contribution of glyphosate itself versus formulation co-components remains unresolved and represents a key uncertainty in the interpretation of glyphosate-associated nephrotoxicity.

### 3.2. Paraquat

#### 3.2.1. Clinical Outcomes

##### Exposure Context and Relevance

Paraquat dichloride is a non-selective bipyridyl herbicide widely used for weed control and crop desiccation in agricultural and non-agricultural settings. Its herbicidal activity is mediated by redox cycling, leading to rapid generation of reactive oxygen species and destruction of plant cell membranes. Extensive use has resulted in diverse opportunities for human exposure, primarily through occupational handling by pesticide applicators and agricultural workers, as well as through environmental residues and, less commonly, dietary intake [32,33]. Acute ingestion of paraquat formulations remains a major cause of severe pesticide poisoning in several regions.

Toxicokinetic studies indicate that paraquat is poorly absorbed following oral expo-sure but, once absorbed, undergoes minimal metabolic transformation and is eliminated largely unchanged via renal excretion. Animal toxicokinetic studies show rapid elimination, with most of the administered dose excreted within 72 h, and renal clearance greater than the glomerular filtration rate indicates active tubular secretion in addition to glomerular filtration [34,35].

##### Acute Kidney Injury in Poisoning Cohorts

Prospective multicenter studies from Sri Lanka indicate that AKI develops early and frequently following paraquat ingestion. In one cohort, 73% of patients fulfilled consensus AKI criteria, with injury becoming apparent within approximately 16–19 h. Serum creatinine increased rapidly in severe cases, often exceeding 100% within 24 h and 300% by 72 h [36] (Supplementary Table S2).

However, serum creatinine appears to overestimate the extent of true glomerular dysfunction during the early phase. Compared with creatinine, serum cystatin C rises more modestly, suggesting that the abrupt creatinine increase reflects not only declining renal function but also oxidative stress-related perturbation of creatine–creatinine metabolism. Accordingly, while serum creatinine is a strong prognostic marker, it is an imperfect surrogate of glomerular filtration in early paraquat toxicity.

Serial urinary biomarker studies further demonstrate early structural tubular injury. In a related multicenter cohort, 76% of patients developed AKI, and urinary cystatin C, NGAL, and clusterin increased within 24 h of ingestion [37]. Albuminuria was also common, occurring in approximately 70% of cases within 16–24 h, and was strongly associated with AKI severity and mortality. Patients with albuminuria exhibited higher concentrations of multiple tubular injury markers, including urinary cystatin C, NGAL, clusterin, KIM-1, and β_2_-microglobulin, supporting a predominant role for tubular dysfunction. Importantly, concurrent proteinuria may inflate urinary biomarker levels and shift diagnostic thresholds, complicating interpretation if not accounted for [38].

The biomarker profile is consistent with oxidative and mitochondrial tubular injury as central features of paraquat-associated AKI. Urinary cytochrome c, a marker of mitochondrial membrane damage, increases early after ingestion and strongly predicts moderate-to-severe AKI, supporting mitochondrial dysfunction as a key event in toxic injury. In contrast, urinary 8-isoprostane shows weaker and less consistent associations, suggesting that lipid peroxidation alone does not fully explain the renal phenotype [23]. Complementary metabolomic evidence further indicates disruption of amino acid and energy metabolism pathways, consistent with impaired mitochondrial bioenergetics and proximal tubular injury [39,40].

Taken together, poisoning cohorts indicate that paraquat-associated AKI is common, develops within the first day after ingestion, and is strongly associated with adverse outcomes. The available evidence supports a model in which early increases in serum creatinine reflect both true renal dysfunction and systemic oxidative stress, whereas albuminuria/proteinuria together with urinary injury biomarkers suggests a mixed pattern of glomerular permeability disturbance and proximal tubular–mitochondrial injury. These findings position AKI as an early and mechanistically informative component of paraquat toxicity.

##### Chronic Kidney Outcomes in Epidemiologic Studies

Evidence linking chronic paraquat exposure to long-term kidney disease is more limited than that for acute poisoning, but several epidemiologic studies suggest a possible association with severe renal outcomes, particularly ESRD (Supplementary Table S2). The strongest individual-level evidence comes from the Agricultural Health Study (AHS), a large prospective cohort of licensed pesticide applicators in the United States. Among male applicators, paraquat use was associated with increased ESRD risk, with elevated hazard ratios observed across the middle and highest tertiles of intensity-weighted cumulative exposure and evidence of an exposure-response trend, although the number of exposed ESRD cases was small [12]. A related AHS analysis among spouses similarly suggested increased ESRD risk associated with indirect exposure, but estimates were imprecise and based on few cases, limiting inference regarding dose–response [41].

Additional, though methodologically weaker, support has been obtained from ecological analyses of county-level paraquat use in the United States, which reported higher ESRD incidence with increasing application density. While broadly consistent with AHS findings, the ecological design precludes individual-level exposure assessment and is inherently susceptible to residual confounding and ecological fallacy [42].

By contrast, not all studies support an association. In a cross-sectional study of agricultural communities in Sri Lanka, urinary paraquat concentrations were not significantly correlated with renal injury biomarkers or measures of kidney function, whereas glyphosate showed associations with several markers. This discrepancy may reflect differences in exposure intensity, timing, or study design, as cross-sectional biomonitoring may inadequately capture cumulative nephrotoxic burden [25].

Overall, the epidemiologic evidence suggests a possible association between chronic paraquat exposure and ESRD, supported primarily by prospective cohort data but limited by small numbers of exposed cases, potential co-exposure to other pesticides, and heterogeneity in exposure assessment and renal endpoints. The principal human evidence for paraquat-related kidney outcomes is summarized in Table 2.

#### 3.2.2. Experimental and Molecular Mechanisms of Nephrotoxicity

Experimental studies in rodents and dogs, together with ex vivo rabbit proximal tubular preparations, consistently identify the renal tubular epithelium, particularly the proximal tubule, as the principal target of paraquat nephrotoxicity. Because paraquat is cleared largely by the kidney through glomerular filtration and active tubular secretion, renal clearance can exceed the glomerular filtration rate, favoring tubular uptake and intracellular accumulation [34,35]. Consistent with this disposition pattern, repeated-dose studies in rodents and dogs have shown dose-dependent renal lesions, including proximal tubular hydropic degeneration, eosinophilia, degeneration, and tubular dilatation [34]. Functional studies in isolated rabbit proximal tubular segments further demonstrated time- and concentration-dependent impairment of organic anion and cation transport, together with reduced oxygen consumption and ouabain-sensitive respiration, indicating that paraquat disrupts tubular transport through mitochondrial energetic failure and secondary inhibition of Na^+^/K^+^-ATPase-dependent transport work [43].

Additional support for early biomarker-detectable renal injury comes from a sublethal male Wistar rat model of paraquat-induced AKI, in which urinary KIM-1, cystatin C, and albumin correlated with histologic renal injury, and KIM-1 showed the best early performance. Together, these findings support early renal injury with a prominent proximal tubular component as a measurable feature of paraquat nephrotoxicity [44].

At the molecular level, classic in vitro and rodent experimental studies indicate that paraquat toxicity is driven primarily by redox cycling, whereby paraquat accepts electrons from intracellular reductases and transfers them to molecular oxygen, generating superoxide and related reactive oxygen species while depleting NADPH and weakening antioxidant defenses [45,46]. The resulting oxidative stress promotes mitochondrial dysfunction, lipid peroxidation, and tubular epithelial injury. This mechanism is further supported by metabolomic findings in patients with acute paraquat intoxication, which showed disturbances in glutamate, glycine, and serine metabolism, consistent with impaired glutathione synthesis and loss of redox homeostasis, as well as alterations in tricarboxylic acid cycle intermediates and other energy-related metabolites that indicate impaired mitochondrial bioenergetics during toxic AKI [39].

Recent data from acute poisoning analyses and experimental cell-based systems suggest that this oxidative injury can progress through regulated stress-response pathways, particularly ferroptosis. Lipidomic and metabolomic analyses in acute paraquat poisoning showed increased polyunsaturated fatty acids and oxidized lipid products, including hydroxyeicosatetraenoic acids (HETEs) and hydroxyoctadecadienoic acid (HODE), together with Fe^2+^ accumulation, glutathione depletion, GPX4 suppression, and a biphasic SLC7A11 response, supporting ferroptosis driven by collapse of the System Xc-/GPX4 antioxidant axis and uncontrolled lipid peroxidation [47]. Ultrastructural findings of mitochondrial swelling, vacuolar degeneration, cristae loss, and outer membrane rupture, together with the greater protective effect of ferrostatin-1 than apoptosis or necroptosis inhibitors, further support this interpretation. Additional mechanistic studies indicate that paraquat nephrotoxicity may also involve mitochondrial ROS–p38–GSDMD pyroptosis and suppression of protective autophagy, suggesting that inflammatory lytic cell death and dysregulated autophagic flux can amplify oxidative tubular injury [48,49].

Overall, the available evidence supports a model in which paraquat nephrotoxicity is a transport-associated tubular injury driven by redox cycling, mitochondrial energetic failure, and lipid peroxidation. These processes promote proximal tubular injury and can progress through ferroptotic, pyroptotic, and autophagy-related stress pathways under conditions of severe oxidative burden. In acute poisoning, such direct tubular toxicity is likely compounded by systemic factors including hypovolemia, circulatory instability, respiratory failure, and metabolic derangement, thereby contributing to the high frequency of AKI observed in severe cases. Under lower-level or nonlethal exposure conditions, however, the dominant pattern may be earlier subclinical tubular dysfunction and biomarker perturbation rather than overt renal failure. Nonetheless, extrapolation to chronic environmental or occupational exposure remains limited by the predominance of acute poisoning models, the relative scarcity of long-term renal studies, and uncertainty regarding the persistence and clinical significance of mild tubular injury after paraquat exposure.

### 3.3. Organophosphates

#### 3.3.1. Clinical Outcomes

##### Exposure Context and Relevance

Organophosphate (OP) insecticides are a major class of agricultural pesticides consisting of pentavalent phosphorus acid esters, including phosphorothionates, phosphorodithioates, and phosphoramidothiolates. Representative compounds include chlorpyrifos, parathion, fenitrothion, dimethoate, malathion, methyl parathion, phorate, terbufos, ethion, acephate, methamidophos, and monocrotophos [50,51].

Human exposure occurs through dietary intake, occupational dermal contact, and inhalation of aerosols or spray drift, with dermal absorption representing the predominant route among agricultural workers [50,52,53]. Following absorption, OPs are widely distributed, with relatively high concentrations reported in the kidney, and are extensively metabolized to dialkyl phosphate (DAP) metabolites, which are commonly used as urinary biomarkers of exposure. Because multiple OPs share common DAP metabolites, these biomarkers reflect class-level exposure rather than specific parent compounds [50,52,54].

Given their widespread use and systemic distribution, increasing attention has been directed toward the potential contribution of OP insecticides to renal injury.

##### Acute Kidney Injury in Poisoning Cohorts

Human evidence linking OP exposure to acute kidney injury (AKI) derives primarily from acute poisoning studies (Supplementary Table S3). In elderly patients with OP poisoning, AKI occurred in approximately 43.7% of cases and correlated with high mortality, together with hypotension and shock, indicating that renal dysfunction is a marker of poisoning severity [55]. In a prospective study of 300 adults, AKI was reported in 22.3% of cases, with delayed presentation associated with increased risk [56].

Compound-specific data show similar patterns. In chlorpyrifos poisoning, AKI occurred in 22.5% of patients and was associated with poor clinical outcomes, with elevated blood urea nitrogen independently predicting adverse prognosis [57]. A nationwide Taiwanese cohort further demonstrated increased subsequent AKI risk following hospitalization for OP/carbamate poisoning, particularly within the first year and among patients requiring mechanical ventilation, although OP-specific effects could not be fully isolated [58].

Case reports provide additional support for direct nephrotoxicity. Malathion inhalation has been associated with nephrotic syndrome and AKI with biopsy evidence of acute tubular necrosis and podocyte injury, while methyl parathion poisoning has been reported to cause reversible AKI in the absence of rhabdomyolysis and classic cholinergic crisis, consistent with a direct tubular mechanism [59,60].

Overall, the available evidence supports a clear association between acute OP poisoning and AKI. However, the relative contributions of direct tubular toxicity and secondary injury due to shock, hypoxia, or multi-organ failure remain difficult to disentangle.

##### Chronic Kidney Outcomes in Epidemiologic Studies

Evidence linking chronic or low-level OP exposure to chronic kidney disease (CKD) is limited and heterogeneous (Supplementary Table S3). Occupational studies have reported associations between pesticide exposure and reduced glomerular filtration or proteinuria, although most involve mixed pesticide exposures and do not isolate OPs [61].

In Thailand, OP/carbamate exposure metrics and erythrocyte acetylcholinesterase inhibition were associated with lower estimated glomerular filtration rate (eGFR), although interpretation was limited by small sample size and single time-point assessment [62].

Biomarker-based studies suggest possible subclinical tubular injury. In a U.S. population analysis, urinary malathion metabolite levels were associated with reduced kidney function, although the cross-sectional design and short half-life of urinary metabolites limit causal inference [63]. In children with CKD, repeated urinary DAP measurements were associated with higher urinary KIM-1 and 8-OHdG, consistent with proximal tubular injury and oxidative stress, but were not consistently associated with longitudinal eGFR decline [64].

Evidence for a role in CKD of unknown etiology (CKDu) remains uncertain. In a case–control study, higher serum OP levels were observed in CKD and CKDu patients, but inverse correlations with GFR suggest that elevated pesticide concentrations may reflect impaired renal clearance rather than causation [65].

Overall, current human evidence supports a strong association between acute OP poisoning and AKI, whereas evidence linking chronic OP exposure to CKD remains limited and methodologically constrained. The principal human evidence for organophosphate insecticide-related kidney outcomes is summarized in Table 3.

#### 3.3.2. Experimental and Molecular Mechanisms of Nephrotoxicity

Experimental evidence indicates that OP nephrotoxicity, as observed across rodent models and renal tubular cell systems, is driven primarily by oxidative stress-mediated proximal tubular injury, with contributions from mitochondrial dysfunction, endoplasmic reticulum (ER) stress, inflammatory signaling, transporter disruption, and apoptotic or necrotic cell death [66].

Early functional studies in male Sprague–Dawley rats demonstrated that diisopropylfluorophosphate (DFP) induces transient diuresis, natriuresis, glucosuria, and proteinuria without hemodynamic changes, supporting a direct tubular mechanism independent of cholinesterase inhibition, with predominant tubular dysfunction and possible glomerular involvement [67]. Repeated methyl parathion exposure in rats similarly increased plasma creatinine and urinary excretion of glucose, phosphate, and albumin, and produced structural tubular injury; these findings are primarily consistent with impaired tubular reabsorptive capacity but also with a possible glomerular permeability component [68].

A large body of animal evidence, largely from rat studies, identifies oxidative stress as a central pathway. OP exposure increases lipid peroxidation and depletes antioxidant defenses, accompanied by elevations in blood urea nitrogen and creatinine and histopathological changes including tubular necrosis, epithelial degeneration, and inflammatory infiltration [69,70,71].

More recent studies indicate that oxidative injury converges on inflammatory and cell-death pathways. In a 28-day male Wistar albino rat model, chlorpyrifos-induced nephrotoxicity was associated with reactive oxygen species generation, HMGB1 release, activation of TLR4/MyD88/NF-κB signaling, suppression of the cytoprotective PPAR-γ/SIRT1 axis, and increased Bax/Bcl-2 ratio with caspase-dependent apoptosis [72]. In a separate 15-day male Sprague–Dawley rat study, chlorpyrifos exposure also increased lipid peroxidation and pro-inflammatory cytokine signaling, accompanied by histopathological lesions, further supporting an oxidative-inflammatory mechanism [73]. Malathion has shown a similarly broad mechanistic profile in experimental rat studies, including activation of ER-stress pathways (ATF6, PERK, IRE1, GRP78, and CHOP); NF-κB-mediated inflammation; apoptosis through Bax, Apaf-1, and caspase-3; and induction of autophagy-related markers such as Beclin-1 and LC3A [74].

Cellular studies further support a proximal tubular target. In HK-2 cells, dichlorvos induced mitochondrial apoptosis through miR-513a-5p upregulation and suppression of Bcl-2, whereas malathion-induced apoptosis was attenuated by miR-96-5p via repression of the ER-stress mediator DDIT3/CHOP [75,76]. These findings suggest that post-transcriptional regulation contributes to OP-induced tubular cell death.

Collectively, the available evidence supports an integrated model in which OP insecticides disrupt tubular epithelial homeostasis primarily through oxidative stress, leading to mitochondrial and ER stress, inflammatory signaling, transporter dysfunction, and apoptotic or necrotic cell death. In acute poisoning, these direct nephrotoxic effects are likely amplified by systemic factors such as shock, respiratory failure, and metabolic derangement. Under repeated or lower-dose exposure conditions, however, the dominant pattern appears to be subclinical proximal tubular injury with biomarker evidence of oxidative stress, rather than overt renal failure. Extrapolation to chronic environmental exposure remains limited by the predominance of high-dose animal models, mixed-exposure epidemiologic designs, and uncertainty regarding compound-specific effects.

### 3.4. Atrazine

#### 3.4.1. Clinical Outcomes

##### Exposure Context and Relevance

Atrazine is a chlorinated triazine herbicide widely used for selective control of grasses and broadleaf weeds, particularly in corn, sorghum, and sugarcane. Human exposure occurs primarily through occupational handling and environmental contamination of surface and groundwater [77,78].

Following absorption, atrazine undergoes extensive biotransformation and is eliminated mainly via urine and feces. Major metabolites include deethylatrazine (DEA), deisopropylatrazine (DIA), diaminochlorotriazine (DACT), and hydroxyatrazine, with dealkylated metabolites predominating in animals [77].

##### Reduced Kidney Function and ESRD in Epidemiologic Studies

Human evidence linking atrazine to kidney outcomes derives mainly from occupational studies (Supplementary Table S4). In the Agricultural Health Study (AHS), a prospective cohort of licensed pesticide applicators in the United States, atrazine exposure showed an exposure-response pattern with incident ESRD, with elevated risks across increasing tertiles of intensity-weighted lifetime use [12]. However, interpretation is limited by the relatively small number of exposed ESRD cases and the potential for residual confounding by correlated pesticide exposures and occupational practices.

Evidence for earlier renal dysfunction is supported by analyses from the Biomarkers of Exposure and Effect in Agriculture (BEEA) study. Among older pesticide applicators, ever use of atrazine was associated with modestly lower estimated glomerular filtration rate (eGFR) and higher odds of CKD, with somewhat stronger associations for recent use [79]. In a subsequent atrazine-focused BEEA analysis, continuous use was associated with lower eGFR and higher serum creatinine and cystatin C, whereas former high users without recent exposure did not show the same pattern, suggesting that ongoing exposure may contribute to subtle impairment of filtration markers [80]. Notably, no consistent association was observed with the tubular injury biomarker KIM-1, which may indicate that the dominant human signal is reduced filtration rather than overt tubular injury at currently observed occupational exposure levels [80].

These findings should nevertheless be interpreted cautiously. In the BEEA analysis, CKD classification relied on a single creatinine or eGFR measurement rather than persistent abnormalities over at least three months, as required by KDIGO criteria [10], and may therefore overestimate established CKD. Even so, the convergence of the prospective AHS ESRD analysis with the cross-sectional BEEA biomarker studies provides moderate human evidence that atrazine exposure may contribute to reduced kidney function and possibly increase ESRD risk in occupationally exposed populations. The principal human evidence for atrazine-related kidney outcomes is summarized in Table 4.

#### 3.4.2. Experimental and Molecular Mechanisms of Nephrotoxicity

Experimental evidence supports the biological plausibility of atrazine-associated nephrotoxicity, with findings from rat and mouse models and selected regulatory dietary toxicology studies centered largely on oxidative stress–related tubular injury, inflammatory signaling, and fibrotic remodeling. Short-term animal studies in Wistar rats have demonstrated increases in serum creatinine, urea, and oxidative stress markers, accompanied by tubular degeneration and activation of antioxidant responses such as the Nrf2 pathway, suggesting redox imbalance as an early event in renal injury [81,82].

Earlier functional studies in rats also suggest that atrazine can impair renal handling before overt renal failure becomes apparent. Subacute exposure increased urinary sodium, potassium, and chloride excretion; induced proteinuria; and reduced creatinine clearance, consistent with altered tubular reabsorption and/or increased glomerular permeability [83]. Although these studies did not define the underlying molecular pathway, they support the view that atrazine can disrupt renal functional integrity at the level of transport and protein handling.

A longer-term exposure model in female Sprague–Dawley rats provides evidence of progressive tubulointerstitial injury, including inflammatory infiltration, tubular atrophy, and fibrosis, often in the absence of marked changes in conventional renal function markers [84]. These findings suggest that structural injury may precede overt changes in serum creatinine or blood nitrogen during chronic low-dose exposure.

Mechanistically, prolonged atrazine exposure in this rat model was associated with increased reactive oxygen species production, upregulation of transforming growth factor-β (TGF-β/TGF-β1), and activation of epithelial–mesenchymal transition (EMT)-related pathways. Increased expression of α-smooth muscle actin, vimentin, fibronectin, matrix metalloproteinase-2, and matrix metalloproteinase-9 was accompanied by activation of Wnt/β-catenin signaling, with increased Wnt and β-catenin expression and suppression of the inhibitory regulators DKK-1 and GSK-3β [84]. These findings support a mechanistic cascade in which sustained oxidative and inflammatory stress promotes EMT-like remodeling and interstitial fibrosis.

Additional work in male Kun-Ming mice suggests that atrazine-induced oxidative injury is closely linked to mitochondrial dysfunction and dysregulated autophagy. In mice, atrazine increased oxidative stress, activated AMPK-dependent autophagy, and altered Nrf2-related responses, with p62 implicated as a key mediator linking autophagy and redox regulation. Lycopene attenuated these changes, supporting a central role for oxidative stress, mitochondrial injury, and maladaptive AMPK-autophagy-Nrf2 cross-talk in atrazine nephrotoxicity [85].

A distinct mechanism identified in regulatory rat and dog dietary studies, as well as in experimental rat studies, involves hydroxyatrazine crystal deposition within renal tubules under high-dose conditions, leading to tubular obstruction and chronic nephropathy. However, this pathway is considered exposure-dependent and its relevance to typical environmental or occupational exposure scenarios remains uncertain [77,86,87].

In summary, the available experimental evidence supports a model in which atrazine exposure is associated with oxidative stress-mediated disruption of tubular epithelial homeostasis, with potential progression toward inflammation and fibrotic remodeling under sustained exposure. Nevertheless, most mechanistic data derive from relatively high-dose animal studies, and extrapolation to human-relevant exposure levels remains limited. These considerations provide biological plausibility for epidemiologic findings of reduced filtration markers and ESRD, while highlighting remaining uncertainties in dose–response relationships and causal interpretation.

## 4. Molecular Signatures of Pesticide-Related Nephrotoxicity

Although the AKI-to-CKD continuum provides a useful translational framework for understanding how acute renal injury may progress to maladaptive repair, fibrosis, and chronic kidney dysfunction, the current pesticide literature does not consistently support a single uniform progression model across compounds. For some pesticides, the strongest human evidence derives from acute poisoning cohorts dominated by AKI, whereas for others the more prominent signal comes from chronic epidemiologic studies showing reduced renal function or CKD-related outcomes. Experimental studies likewise identify tubular injury, oxidative stress, inflammatory signaling, mitochondrial dysfunction, and fibrosis-related pathways, but these findings do not always map onto a continuous longitudinal sequence in humans. Accordingly, across the pesticides reviewed, nephrotoxicity is better understood as a set of partially overlapping renal molecular signatures rather than a single nonspecific oxidative-stress pathway [30,47,72,84]. The most consistent cross-pesticide lesion is proximal tubular epithelial injury, in which oxidative imbalance, mitochondrial dysfunction, transporter disruption, and early biomarker release often precede major changes in serum creatinine or estimated glomerular filtration rate [20,38,64]. However, the dominant downstream programs differ across pesticide classes: glyphosate-based herbicides are most strongly characterized by oxidative tubular apoptosis, Ca^2+^-linked stress signaling, and membrane/ATPase dysfunction [29,30]; atrazine by oxidative tubular dysfunction with progressive inflammatory-fibrotic remodeling, with an additional high-dose hydroxyatrazine crystal-nephropathy mechanism [77,87]; paraquat by transport-mediated proximal tubular injury driven by redox cycling, mitochondrial energetic failure, and lipid peroxidation, with emerging evidence for ferroptotic and other stress-response pathways [35,39,47,48]; and organophosphate insecticides by oxidative-inflammatory tubular injury coupled to ER stress and apoptosis [72,74].

**Figure 2:**
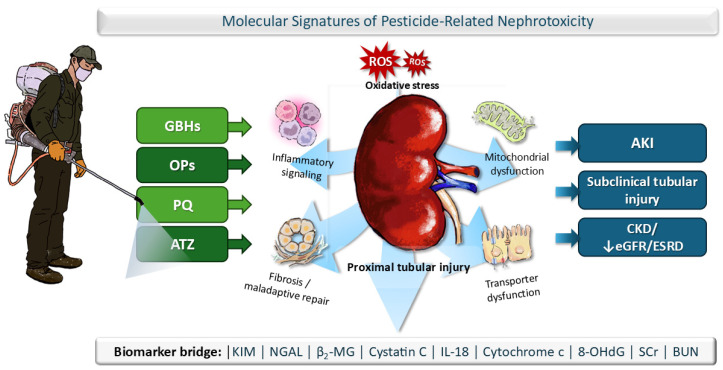
Different pesticide classes converge on proximal tubular epithelial injury through partially overlapping molecular signatures, including oxidative stress, mitochondrial dysfunction, transporter disruption, inflammatory signaling, and maladaptive repair, which together contribute to AKI, subclinical tubular injury, and CKD-related outcomes. (ATZ, atrazine; GBHs, glyphosate-based herbicides; OP, organophosphate; PQ, paraquat).

From a translational perspective, these signatures suggest that pesticide-associated renal injury may be underestimated when assessment relies solely on conventional functional indices [37,38,79,80]. Biomarkers that map more directly onto tubular biology and oxidative injury—including KIM-1, NGAL, β_2_-microglobulin, cystatin C, IL-18, cytochrome c, and 8-OHdG—appear especially informative because they capture early epithelial stress, impaired tubular handling, mitochondrial injury, and oxidative DNA damage before overt renal failure becomes apparent [20,25,27,64,88] (Figure 2). Thus, although the mechanistic literature is heterogeneous in depth and quality, a cross-pesticide synthesis supports the existence of biologically coherent renal injury phenotypes that strengthen causal plausibility for the epidemiologic associations summarized in this review. The principal molecular signatures, representative markers, major renal phenotypes, and overall strength of support are summarized in Table 5.

## 5. Conclusions

Current evidence indicates that pesticide-related nephrotoxicity is mechanistically heterogeneous but exhibits a coherent pattern at the level of target tissue, with consistent involvement of the proximal tubule across multiple pesticide classes. While glyphosate-based herbicides, atrazine, paraquat, and organophosphate insecticides differ in their primary modes of action, the convergence of human, experimental, and biomarker data supports a common pattern of early tubular vulnerability that may not be fully captured by conventional renal function indices.

The human evidence is strongest for acute kidney injury following severe poisoning, particularly for glyphosate-based herbicides, paraquat, and organophosphates. In contrast, evidence linking chronic occupational or environmental exposure to CKD or ESRD remains limited and heterogeneous, reflecting challenges in exposure characterization, co-exposures, and outcome assessment. Nevertheless, the overall concordance across epidemiologic studies, experimental models, and mechanistically informative biomarkers suggests that pesticide-associated renal injury may often begin as subclinical tubular dysfunction before overt clinical disease becomes apparent.

From a regulatory perspective, these findings highlight important gaps in current toxicological assessment frameworks. Standard toxicity studies are effective in identifying overt nephrotoxicity but are less sensitive to early or subclinical renal injury, particularly under exposure scenarios relevant to poisoning or chronic low-level exposure. Incorporation of mechanistically anchored biomarkers, especially those reflecting tubular injury and dysfunction, may improve the detection and characterization of early nephrotoxic effects when used alongside conventional endpoints.

This review should also be interpreted in light of several limitations. Human literature remains constrained by heterogeneous exposure assessment, frequent co-exposures to other pesticides or environmental stressors, and substantial reliance on poisoning cohorts for some compounds, particularly when evaluating AKI. For chronic exposure settings, many studies were cross-sectional, limiting causal inference and making it difficult to distinguish transient functional changes from persistent kidney disease. In addition, although early tubular biomarkers provide biologically informative signals of renal stress and injury, their relationship to long-term clinical outcomes such as CKD progression or ESRD remains incompletely defined.

Future research should prioritize improved exposure assessment, longitudinal study designs, and integration of early biomarkers with clinically meaningful outcomes. Such approaches are needed to distinguish transient toxic injury from persistent renal dysfunction and to clarify the extent to which subclinical tubular effects translate into long-term kidney disease. Progress toward a more mechanism-informed and exposure-relevant framework will be critical for refining the assessment and management of pesticide-related nephrotoxicity.

## Figures and Tables

**Table 1:** Human evidence for kidney effects of glyphosate and glyphosate-based herbicides.

Context	Renal Signal	Marker Pattern	Evidence
Acute GBH poisoning	AKI; early tubular injury	↑ urinary IL-18, NGAL, TFF3, cystatin C, and cytochrome c before/with AKI	Moderate [18,20,23,24]
High surfactant burden	More severe AKI	Surfactant load predicts AKI and complications better than glyphosate amount	Limited to moderate [18]
Occupational/agricultural exposure	Mild chronic renal signal	↓ eGFR with ↑ ACR, NGAL, β_2_-microglobulin, and cystatin C	Limited [11,25]
Environmental exposure	Subclinical tubular signal or null findings	↑ KIM-1 despite normal eGFR in some studies, but no clear association in others	Limited [26,27]

Abbreviations: ACR, albumin-to-creatinine ratio; AKI, acute kidney injury; eGFR, estimated glomerular filtration rate; GBH, glyphosate-based herbicide; IL-18, interleukin-18; KIM-1, kidney injury molecule-1; NGAL, neutrophil gelatinase-associated lipocalin; TFF3, trefoil factor 3. ↑ indicates increased levels or activity; ↓ indicates decreased levels or activity.

**Table 2:** Human Evidence for Kidney Effects of Paraquat.

Context	Renal Signal	Marker Pattern	Evidence
Acute poisoning	Early AKI	Creatinine rises more steeply than cystatin C	Strong [36]
Serial biomarker studies	Early structural tubular injury	↑ urinary cystatin C, NGAL, and clusterin within 24 h	Strong [37]
Albuminuric AKI	More severe glomerular permeability disturbance and tubular dysfunction	Albuminuria tracks AKI severity and mortality and is associated with higher urinary injury biomarkers	Strong [38]
Acute biomarker evidence	Mitochondrial tubular injury in AKI	↑ cytochrome c in acute AKI	Moderate [23,39]
Chronic occupational exposure	Possible ESRD risk	Exposure-response signal in AHS, with weaker or less precise support in other studies	Moderate [12,41,42]

Abbreviations: AHS, Agricultural Health Study; AKI, acute kidney injury; ESRD, end-stage renal disease; NGAL, neutrophil gelatinase-associated lipocalin. ↑ indicates increased levels or activity.

**Table 3:** Human Evidence for Kidney Effects of Organophosphate Insecticides.

Context	Renal Signal	Marker Pattern	Evidence
Acute OP poisoning	AKI	AKI more frequent with severe poisoning, shock, and delayed presentation	Strong [55,56,57,58]
Compound-specific poisoning	Direct nephrotoxicity	↑ creatinine/BUN; ATN and occasional podocyte injury in case reports	Limited to moderate [57,59,60]
Occupational/mixed exposure	Reduced kidney function	↓ eGFR and ↑ proteinuria/cholinesterase inhibition in some studies	Limited [61,62]
Repeated biomonitoring	Subclinical tubular/oxidative injury	↑ KIM-1 and 8-OHdG, with lower baseline eGFR, but no consistent CKD progression	Limited to moderate [63,64,65]

Abbreviations: AKI, acute kidney injury; ATN, acute tubular necrosis; BUN, blood urea nitrogen; CKD, chronic kidney disease; eGFR, estimated glomerular filtration rate; KIM-1, kidney injury molecule-1; OP, organophosphate; 8-OHdG, 8-hydroxy-2′-deoxyguanosine. ↑ indicates increased levels or activity; ↓ indicates decreased levels or activity.

**Table 4:** Human Evidence for Kidney Effects of Atrazine.

Context	Renal Signal	Marker Pattern	Evidence
Occupational cohort	ESRD risk	Positive exposure-response pattern in AHS	Moderate [12]
AHS/BEEA biomonitoring	Reduced kidney function	↓ eGFR and ↑ CKD odds, especially in recent users	Limited to moderate [79]
Continuous-use biomonitoring	Subclinical renal dysfunction	↓ eGFR with ↑ creatinine/cystatin C; no clear KIM-1 signal	Limited to moderate [80]

Abbreviations: AHS, Agricultural Health Study; BEEA, Biomarkers of Exposure and Effect in Agriculture; CKD, chronic kidney disease; eGFR, estimated glomerular filtration rate; ESRD, end-stage renal disease; KIM-1, kidney injury molecule-1. ↑ indicates increased levels or activity; ↓ indicates decreased levels or activity.

**Table 5:** Principal Molecular Signatures of Pesticide-Related Nephrotoxicity.

Signature	Marker/Pathway Pattern	Renal Meaning	Best-Supported Pesticides	Support
**Oxidative stress/redox imbalance**	↑ ROS, lipid peroxidation, 8-OHdG, MDA; antioxidant depletion	Early tubular stress and injury	GBHs, PQ, OPs, ATZ [28,46,72,84]	Strong
**Mitochondrial injury/apoptosis**	↑ cytochrome c, Bax/Bcl-2 imbalance, caspase activation; impaired bioenergetics	Tubular epithelial apoptosis; AKI	GBHs, PQ; supportive evidence in OPs [20,23,30,39]	Strong
**Proximal tubular injury/dysfunction**	↑ KIM-1, NGAL, β_2_-microglobulin, cystatin C, IL-18, clusterin	Early tubular injury with impaired reabsorption	GBHs, PQ, OPs [20,25,27,38,64]	Strong
**Transporter/membrane dysfunction**	ATPase inhibition, altered tubular transport, glucosuria, natriuresis, proteinuria	Functional tubular impairment, with possible glomerular permeability disturbance in proteinuric states	GBHs, PQ, OPs; supportive evidence in ATZ [29,30,43,83]	Moderate to strong
**Inflammatory/ER-stress signaling**	HMGB1/TLR4/NF-κB, cytokine induction, ATF6/PERK/IRE1/CHOP	Tubulointerstitial inflammation and injury amplification	OPs, ATZ; supportive evidence in PQ [72,74,84]	Moderate
**Fibrosis/maladaptive repair**	TGF-β, fibronectin, collagen, EMT-related pathways, Wnt/β-catenin	Chronic tubulointerstitial remodeling and fibrosis	ATZ; more limited support in prolonged injury models of other pesticides [84]	Moderate
**Ferroptotic/lipid peroxidation-driven injury**	Oxidized lipids, Fe^2+^ accumulation, GPX4 suppression, SLC7A11 dysregulation	Severe oxidative tubular injury	PQ [47,48]	Limited to moderate
**Crystal-related tubular obstruction**	Hydroxyatrazine crystal deposition	Tubular obstruction and crystal nephropathy	ATZ [77,87]	Limited but specific

Abbreviations: 8-OHdG, 8-hydroxy-2′-deoxyguanosine; AKI, acute kidney injury; ATZ, atrazine; EMT, epithelial–mesenchymal transition; ER, endoplasmic reticulum; GBHs, glyphosate-based herbicides; IL-18, interleukin-18; KIM-1, kidney injury molecule-1; MDA, malondialdehyde; NF-κB, nuclear factor kappa B; NGAL, neutrophil gelatinase-associated lipocalin; OPs, organophosphate insecticides; PQ, paraquat; ROS, reactive oxygen species; TGF-β, transforming growth factor beta. ↑ indicates increased levels or activity.

## Data Availability

No new data were created or analyzed in this study. Data sharing is not applicable to this article.

## Supplementary Materials

The following supporting information can be downloaded at https://www.mdpi.com/article/10.3390/ijms27093970/s1. References [89,90,91,92,93,94] are cited only in the Supplementary Materials.

## Abbreviations

The following abbreviations are used in this manuscript:

8-OHdG	8-hydroxy-2′-deoxyguanosine
AHS	Agricultural Health Study
AKI	acute kidney injury
AKIN	Acute Kidney Injury Network
AMPA	aminomethylphosphonic acid
AMPK	AMP-activated protein kinase
Apaf-1	apoptotic protease-activating factor 1
ATF6	activating transcription factor 6
ATN	acute tubular necrosis
ATZ	atrazine
AUC-ROC	area under the receiver operating characteristic curve
Bax	Bcl-2-associated X protein
Bcl-2	B-cell lymphoma 2
BEEA	Biomarkers of Exposure and Effect in Agriculture
CHOP	C/EBP homologous protein
CKD	chronic kidney disease
CKDu	chronic kidney disease of unknown etiology
DACT	diaminochlorotriazine
DAP	dialkyl phosphate
DEA	deethylatrazine
DDIT3	DNA damage-inducible transcript 3
DFP	diisopropylfluorophosphate
DIA	deisopropylatrazine
DKK-1	dickkopf-related protein 1
eGFR	estimated glomerular filtration rate
EFSA	European Food Safety Authority
EMT	epithelial–mesenchymal transition
EPA	U.S. Environmental Protection Agency
ER	endoplasmic reticulum
ESKD	end-stage kidney disease
ESRD	end-stage renal disease
GBH	glyphosate-based herbicide
GPSH	glyphosate surfactant herbicide
GPX4	glutathione peroxidase 4
GRP78	glucose-regulated protein 78
GSDMD	gasdermin D
GSK-3β	glycogen synthase kinase-3 beta
HbA1c	glycated hemoglobin
HETEs	hydroxyeicosatetraenoic acids
HK-2	human kidney-2 cells
HMGB1	high-mobility group box 1
HODE	hydroxyoctadecadienoic acid
IL-18	interleukin-18
IRE1	inositol-requiring enzyme 1
JMPR	Joint FAO/WHO Meeting on Pesticide Residues
KDIGO	Kidney Disease: Improving Global Outcomes
KIM-1	kidney injury molecule-1
LC3A	microtubule-associated protein 1 light chain 3 alpha
MeSH	Medical Subject Headings
MyD88	myeloid differentiation primary response 88
NADPH	nicotinamide adenine dinucleotide phosphate, reduced form
NF-κB	nuclear factor kappa B
NGAL	neutrophil gelatinase-associated lipocalin
NMDAR	N-methyl-D-aspartate receptor
Nrf2	nuclear factor erythroid 2-related factor 2
OP	organophosphate
PQ	paraquat
PERK	protein kinase RNA-like endoplasmic reticulum kinase
PPAR-γ	peroxisome proliferator-activated receptor gamma
RIFLE	Risk, Injury, Failure, Loss, and End-stage kidney disease
RRT	renal replacement therapy
ROS	reactive oxygen species
SIRT1	sirtuin 1
SLC7A11	solute carrier family 7 member 11
system Xc−	cystine/glutamate antiporter
TFF3	trefoil factor 3
TGF-β	transforming growth factor beta
TLR4	Toll-like receptor 4
USRDS	United States Renal Data System
WHO/IPCS	World Health Organization/International Programme on Chemical Safety
Wnt/β-catenin	Wingless/integrated–beta-catenin signaling pathway
