# Supplementary material for: Evidence-Based Assessment of Pesticide-Related Nephrotoxicity: Clinical Outcomes, Experimental Data, and Molecular Signatures
Source: Int J Mol Sci. 2026 Apr 29;27(9):3970. doi: 10.3390/ijms27093970 (PMC13163954; doi:10.3390/ijms27093970)
Supplement: Supplementary file 1 [file ijms-27-03970-s001.zip › ijms-4276484-supplementary.pdf]

Supplementary Table S1: Glyphosate, glyphosate-based herbicides (GBHs) and kidney outcomes

Study	Exposure setting	Population	Kidney outcome	Main finding	Strength / interpretation
**Seok et al., 2011 [18]**	Acute glyphosate-surfactant intoxication	107 poisoned patients	AKI	AKI occurred in 17.1%; surfactant volume predicted AKI and other complications better than glyphosate ingredient volume.	**Limited to moderate** evidence that acute renal toxicity is driven largely by formulation surfactant load.
**Garlich et al., 2014 [19]**	Acute poisoning case report	1 patient	Severe AKI	Severe AKI with acidosis and hyperkalemia improved after hemodialysis.	**Weak or insufficient** evidence; supportive case report only.
**Mohamed et al., 2016 [20]**	Acute GBH poisoning	Multicentre poisoning cohort	AKI; early tubular injury	Early urinary IL-18, cytochrome c, NGAL, TFF3, and cystatin C predicted moderate–severe AKI, consistent with proximal tubular injury.	**Moderate to strong** acute poisoning evidence; supports tubular/mitochondrial toxicity, but mechanism remains formulation-specific.
**Wijerathna et al., 2020 [23]**	Acute GBH poisoning biomarker study	GBH subgroup within toxic-AKI cohort	AKI; mitochondrial tubular injury	Urinary cytochrome c rose early and predicted moderate–severe AKI; 8-isoprostane was not useful.	**Moderate** mechanistic evidence for mitochondrial tubular injury in acute poisoning.
**Cho et al., 2023 [24]**	Acute GBH poisoning	184 patients	AKI	AKI occurred in 44.5% and was associated with hypotension, ECG abnormalities, longer hospitalization, and higher mortality.	**Moderate** clinical evidence that AKI is common and prognostically important after acute poisoning.
**Jayasumana et al., 2015 [11]**	Environmental / occupational exposure	125 CKDu cases, 180 controls	CKDu	Glyphosate use was associated with CKDu after adjustment, alongside well-water and abandoned-well exposures.	**Limited** positive signal: interpretation is constrained by strong co-exposure and confounding concerns.

Study	Exposure setting	Population	Kidney outcome	Main finding	Strength / interpretation
**Lebov et al., 2016 [12]**	Occupational exposure	24,429 male pesticide applicators	ESRD	No clear association was found between glyphosate exposure and ESRD.	**Limited** evidence against a major chronic ESRD effect in this cohort.
**Trasande et al., 2020 [26]**	Low-level environmental exposure	108 infants and young children	KIM-1, NGAL, albuminuria	No significant association was found between urinary glyphosate and renal injury biomarkers.	**Weak or insufficient** evidence for nephrotoxicity at low environmental exposure in this population.
**Abdul et al., 2021 [25]**	Occupational biomonitoring	348 sugarcane farmers screened: 210 sampled	Reduced eGFR; increased ACR; tubular injury biomarkers	Urinary glyphosate correlated with lower eGFR, higher ACR, NGAL, β_2-microglobulin, and serum cystatin C.	**Limited** cross-sectional evidence for early chronic renal injury.
**Romo-García et al., 2025 [27]**	Environmental biomonitoring	221 children	KIM-1; eGFR	Higher urinary glyphosate was associated with higher KIM-1, while eGFR remained normal.	**Limited** evidence for subclinical tubular injury, not established renal dysfunction.

Abbreviations: GBHs, glyphosate-based herbicides; AKI, acute kidney injury; CKDu, chronic kidney disease of unknown etiology; ESRD, end-stage renal disease; KIM-1, kidney injury molecule-1; NGAL, neutrophil gelatinase-associated lipocalin; eGFR, estimated glomerular filtration rate; ACR, albumin-to-creatinine ratio; IL-18, interleukin-18; TFF3, trefoil factor 3; ECG, electrocardiography.

Supplementary Table S2: Paraquat and kidney outcomes

Study	Exposure setting	Population	Kidney outcome	Main finding	Strength / interpretation
**Mohamed, Endre, et al., 2015 [36]**	Acute self-poisoning cohort	66 patients with acute paraquat poisoning	AKI	AKI developed in 73%; creatinine rose much faster than cystatin C, indicating early creatinine inflation beyond true GFR loss.	**Strong** acute clinical evidence for AKI; moderate mechanistic evidence that oxidative stress distorts creatinine kinetics.
**Mohamed, Buckley, et al., 2015 [37]**	Acute self-poisoning cohort with serial biomarkers	50 paraquat-poisoned patients	AKI; early tubular injury	Functional AKI occurred in 76%; urinary cystatin C, NGAL, and clusterin rose within 24 h and identified early tubular injury.	**Strong** evidence for acute paraquat-associated AKI with an early tubular injury signal.
**Mohamed et al., 2017 [38]**	Acute self-poisoning cohort; albuminuria-focused analysis	50 confirmed paraquat-poisoned patients	AKI; albuminuria-associated glomerular and tubular injury	Albuminuria was common, increased with AKI severity, and tracked mortality; higher urinary injury biomarkers in albuminuric patients suggested mixed glomerular and tubular involvement, although some cases may have had pre-existing albuminuria.	**Strong** evidence for acute nephrotoxicity; **moderate to stron**g evidence that albuminuria/proteinuria reflects mixed glomerular and tubular involvement and influences biomarker interpretation.
**Wan et al., 2019 [39]**	Acute paraquat intoxication hospital study	40 patients with acute paraquat intoxication; 10 controls	AKI	AKI occurred in 20/40 patients and was linked to higher paraquat burden, illness severity, cystatin C, BUN, creatinine, and metabolomic abnormalities.	**Moderate** evidence for acute AKI, with supportive metabolomic evidence for tubular/metabolic injury.

Study	Exposure setting	Population	Kidney outcome	Main finding	Strength / interpretation
**Wijerathna et al., 2020 [23]**	Acute poisoning biomarker cohort	Paraquat subgroup n=21 within a multicenter toxic-AKI cohort	AKI; mitochondrial tubular injury	Urinary cytochrome c strongly predicted moderate–severe AKI, whereas 8-isoprostane did not.	**Moderate** mechanistic evidence for mitochondrial tubular injury in acute paraquat AKI.
**Lebov et al., 2015 [41]**	Para-occupational cohort study	31,142 wives of pesticide applicators; 98 ESRD cases	ESRD	Indirect paraquat exposure through husbands' use was associated with higher ESRD risk.	**Limited to moderate** evidence for para-occupational ESRD risk, but estimates are imprecise.
**Lebov et al., 2016 [12]**	Occupational cohort study	24,429 male pesticide applicators; 320 ESRD cases	ESRD	Higher paraquat exposure tertiles were associated with increased ESRD risk, with a positive exposure-response trend.	**Moderate** evidence for chronic occupational ESRD risk.
**Abdul et al., 2021 [25]**	Community-based cross-sectional biomonitoring study	210 farmers in Sri Lanka	Reduced kidney function; tubular injury biomarkers	Urinary paraquat did not significantly correlate with eGFR, ACR, NGAL, or KIM-1.	**Limited** evidence against a consistent paraquat-specific chronic biomarker association in this dataset.
**McGwin & Griffin, 2022 [42]**	Ecological environmental exposure study	County-level analysis in the conterminous USA	ESRD	ESRD incidence increased across higher county paraquat-use quintiles.	**Limited to moderat**e evidence; ecologic design supports a population-level signal but not individual-level causation.

Abbreviations: AKI, acute kidney injury; ESRD, end-stage renal disease; eGFR, estimated glomerular filtration rate; ACR, albumin-to-creatinine ratio; NGAL, neutrophil gelatinase-associated lipocalin; KIM-1, kidney injury molecule-1; BUN, blood urea nitrogen; GFR, glomerular filtration rate.

Supplementary Table S3: Organophosphates (OPs) and kidney outcomes

Study	Exposure setting	Population	Kidney outcome	Main finding	Strength / interpretation
**Vikrant, 2015 [59]**	Acute methyl parathion inhalation	1 patient	AKI; hepatorenal toxicity	Reversible AKI developed after methyl parathion exposure, with elevated creatinine and depressed pseudocholinesterase supporting OP toxicity.	**Limited** evidence; supportive case report for reversible tubular injury.
**Lee et al., 2015 [58]**	Acute OP/carbamate poisoning	8,924 poisoned patients; 35,691 matched controls	AKI	OP/carbamate poisoning was associated with a markedly increased subsequent AKI risk (adjusted HR 6.17, 95% CI 3.28–11.6), especially within the first year and in severe cases.	**Strong** evidence for acute class-level nephrotoxicity after cholinesterase-inhibiting pesticide poisoning, though OPs and carbamates were not fully separated.
**Yokota et al., 2017 [60]**	Acute malathion inhalation	1 patient	AKI; nephrotic syndrome	Biopsy showed acute tubular necrosis with podocyte injury after malathion exposure, with recovery after dialysis/steroids.	**Limited** evidence; mechanistically informative case report only.
**Liu et al., 2020 [57]**	Acute chlorpyrifos intoxication	40 poisoned patients	AKI	Kidney injury occurred in 22.5% of chlorpyrifos poisonings and was more frequent in the poor-prognosis group.	**Moderate** evidence for renal involvement in severe acute chlorpyrifos poisoning; limited by small, single-center retrospective design.

Study	Exposure setting	Population	Kidney outcome	Main finding	Strength / interpretation
**Yu et al., 2021 [55]**	Acute OP poisoning in elderly patients	71 elderly patients with OP poisoning	AKI	AKI occurred in 43.7% of elderly OP-poisoned patients and was significantly more frequent in nonsurvivors, alongside hypotension and shock.	**Moderate** evidence that AKI is a common and prognostically adverse complication of severe acute OP poisoning in older adults; limited by retrospective single-cohort design.
**Hanif & Sattar, 2022 [56]**	Acute OP poisoning survey	300 adults presenting within 24 h of OP poisoning	AKI	AKI frequency was 22.3% and was lower in patients presenting earlier after poisoning.	**Limited to moderate** descriptive evidence that AKI is a frequent early complication of acute OP poisoning; limited by cross-sectional design and creatinine-based case definition.
**Prudente et al., 2021 [61]**	Occupational agricultural exposure	208 citrus farmers	Reduced renal function	A substantial proportion of workers showed reduced GFR/proteinuria, and longer pesticide exposure was associated with higher risk of impaired renal function.	**Limited to moderate** evidence for occupational renal impairment, but exposure was mixed and cross-sectional.
**Sombatsawat et al., 2021 [62]**	Occupational OP/carbamate exposure	58 Thai farmers	Reduced kidney function / CKDu-like phenotype	Cholinesterase inhibition was associated with lower eGFR, and OP/carbamate exposure was associated with reduced renal function (OR 4.70, 95% CI 1.02–21.69).	**Limited** evidence; suggestive signal only, constrained by small sample size, no unexposed group, and single creatinine measurement.

Study	Exposure setting	Population	Kidney outcome	Main finding	Strength / interpretation
**Wan et al., 2021 [63]**	General-population biomonitoring	3,557 participants with metabolite data within NHANES	Reduced kidney function	Urinary malathion metabolite levels were associated with higher odds of low kidney function (aOR 1.26, 95% CI 1.01–1.56; sensitivity analysis 1.32, 95% CI 1.01–1.73).	**Limited to moderate** evidence for an association between malathion exposure and reduced kidney function in the general population.
**Jacobson et al., 2021 [64]**	Repeated biomonitoring in established CKD	618 children with mild-to-moderate CKD	Lower eGFR at baseline; subclinical tubular injury	Higher urinary OP metabolites, especially diethyl metabolites, were associated with lower baseline eGFR, higher KIM-1, and higher 8-OHdG, but not with consistent CKD progression.	**Moderate** evidence for subclinical tubular/oxidative injury; **limited** evidence for clinically meaningful CKD progression.
**Atlani et al., 2025 [65]**	Case-control study in CKDu/CKD region	55 CKDu, 53 CKD, 50 healthy controls	CKDu / CKD	Higher serum chlorpyrifos and ethion were seen in CKDu and CKD versus controls, but similar elevations in CKDu and CKD and inverse correlation with GFR raise concern for reverse causation.	**Limited** evidence linking OPs to CKDu; pesticide accumulation may reflect impaired renal clearance rather than causation.

Abbreviations: OPs, organophosphates; AKI, acute kidney injury; CKD, chronic kidney disease; CKDu, chronic kidney disease of unknown etiology; eGFR, estimated glomerular filtration rate; HR, hazard ratio; CI, confidence interval; OR, odds ratio; aOR, adjusted odds ratio; KIM-1, kidney injury molecule-1; 8-OHdG, 8-hydroxy-2′-deoxyguanosine; NHANES, National Health and Nutrition Examination Survey.

Supplementary Table S4: Atrazine and kidney outcomes

Study	Exposure setting	Population	Kidney outcome	Main finding	Strength / interpretation
**Lebov et al., 2016 [12]**	Occupational cohort study	Male licensed pesticide applicators in Iowa and North Carolina; 320 ESRD cases during follow-up	ESRD	Highest tertile of intensity-weighted atrazine use was associated with increased ESRD risk versus non-use (HR 1.52, 95% CI 1.11–2.09), with a positive exposure-response trend.	**Moderate** evidence for a chronic ESRD association in occupationally exposed applicators; interpretation is tempered by limited covariate adjustment.
**Shearer et al., 2021 [79]**	Occupational biomonitoring / cross-sectional AHS subcohort	1,545 older male pesticide applicators	Reduced kidney function; CKD	Ever use of atrazine was associated with lower eGFR and higher odds of CKD; associations were stronger in recent users, with evidence of an exposure–response pattern.	**Limited to moderate** evidence for reduced kidney function associated with atrazine use; limited by cross-sectional design and single-time-point CKD classification.
**Andreotti et al., 2025 [80]**	Occupational biomonitoring / molecular epidemiology study	Male pesticide applicators ≥50 years old; continuous users n=264, controls n=246	Reduced kidney function; subclinical glomerular dysfunction	Continuous atrazine users had lower eGFR and higher serum creatinine and cystatin C than never/low users, while former high users showed no clear association.	**Limited to moderate** evidence for early subclinical glomerular dysfunction associated with ongoing atrazine exposure.

Abbreviations: ESRD, end-stage renal disease; CKD, chronic kidney disease; eGFR, estimated glomerular filtration rate; AHS, Agricultural Health Study; HR, hazard ratio; CI, confidence interval.

Supplementary Table S5. Experimental models and study descriptors supporting mechanistic interpretation of pesticide-related nephrotoxicity

Pesticide / class	Study	Model / species	Exposure route	Dose / concentration	Duration	Key renal / mechanistic finding
**Glyphosate / GBHs**	**Mesnage et al., 2015 [89]**	Female rats, 10/group	Drinking water	GBH 0.1 ppb (50 ng/L glyphosate equivalent; estimated daily intake 4 ng/kg bw/day)	2 years	Ultra-low-dose GBH altered blood/urine biochemistry consistent with impaired kidney function and induced kidney transcriptomic changes linked to fibrosis, necrosis, mitochondrial dysfunction, ischemia, and altered mTOR-related signaling. **Note:** Included as supportive background only; interpretation is limited by the small group size, female-only transcriptomic analysis after most male tissues became unsuitable because of postmortem necrosis, and reliance on indirect biochemical/transcriptomic renal signals without marked anatomical kidney injury.
	**Hamdaoui et al., 2016 [28]**	Female Wistar rats	Oral gavage	126 or 315 mg/kg glyphosate-equivalent (GBH)	60 days	GBH exposure increased plasma creatinine, urea, and uric acid, reduced creatinine clearance, induced oxidative stress, and caused proximal/distal tubular necrosis with renal inflammation.

Pesticide / class	Study	Model / species	Exposure route	Dose / concentration	Duration	Key renal / mechanistic finding
	**Dedeke et al., 2018 [29]**	Adult male rats	Oral gavage	3.6, 50.4, or 248.4 mg/kg bw glyphosate-equivalent (GBH vs glyphosate alone)	12 weeks	GBH but not glyphosate alone, caused marked oxidative stress, ATPase suppression, increased renal glyphosate accumulation, elevated NGAL and renal function markers, and dose-dependent glomerular and tubular injury.
	**Gao et al., 2019 [30]**	HK-2 cells	*In vitro*	Glyphosate 0, 20, 40, 60 μM	24 h	Glyphosate induced proximal tubular apoptosis through an NMDAR–Ca^{2+}–ROS pathway in HK-2 cells and caused early urinary low-molecular-weight protein changes, tubular cell exfoliation, apoptosis, and renal oxidative stress in mice
		ICR mice	Oral gavage	Glyphosate 400 mg/kg/day	28 days	
	**Turkmen et al., 2019 [90]**	Male Wistar rats	Oral gavage	GBH 375 mg/kg/day± NAC 160 mg/kg	8 weeks	GBH increased urea and creatinine, induced oxidative stress, and caused tubular epithelial cells and vacuolar/glomerular changes.; these changes were attenuated by N-acetylcysteine.
	**Gadotti et al., 2023 [31]**	Male Wistar rats	Oral gavage	GBH 0, 0.5, or 5 mg/kg	From PND23 to PND60 or PND90	Low-dose GBH produced mild but coherent tubulointerstitial injury, with tubular/interstitial swelling, inflammatory infiltration, increased *Havcr1*(*Kim1*), fibrosis at the higher dose, and altered renal transporter-gene expression.

Pesticide / class	Study	Model / species	Exposure route	Dose / concentration	Duration	Key renal / mechanistic finding
	**Nacano et al., 2024 [91]**	Male Wistar rats	Oral gavage	Glyphosate 5 mg/kg bw/day	25 days dosing; terminal assessment at day 30	Early renal injury marked by ↓ kidney weight, proximal tubular vacuolation, ↑ KIM-1, and increased urinary oxidative stress, with limited change in conventional renal function markers.
	**Chukwubueze et al., 2025 [92]**	Sprague–Dawley rats; both sexes	Oral gavage	GBH 100 mg/kg bw/day	12 weeks	Kidney glycomic/proteomic remodeling with inflammatory and oxidative-stress signaling, including ↑ fucosylated/sialofucosylated N-glycans, immune pathway activation, and stronger female susceptibility.
**Paraquat**	**Bus et al., 1976 [45]**	Mouse lung microsomes	*In vitro*	PQ 10^{-6}–10^{-4} M	Acute incubation	NADPH-dependent PQ redox cycling increased MDA formation; inhibition by superoxide dismutase and a singlet oxygen trap supported superoxide-/singlet oxygen-mediated lipid peroxidation.
		Mice	IP; plus dietary/oxygen manipulations	PQ 30 mg/kg IP; phenobarbital pretreatment; selenium or vitamin E deficiency; diethyl maleate pretreatment; 100% O_2 exposure	Acute to 7 days; antioxidant measurements up to 193 h	Hyperoxia and antioxidant deficiency (selenium, vitamin E, or GSH depletion) increased PQ lethality, while sustained phenobarbital pretreatment reduced toxicity; PQ also depleted liver GSH and lung lipid-soluble antioxidants.

Pesticide / class	Study	Model / species	Exposure route	Dose / concentration	Duration	Key renal / mechanistic finding
		Rats	Drinking water or IP	PQ 100 ppm in drinking water for 3 weeks; or PQ 45 mg/kg IP after 85% O_2 pretreatment	3 weeks or acute survival study	Chronic PQ exposure increased pulmonary GSH reductase and G6PD activity, and oxygen-tolerant rats showed prolonged survival after PQ, consistent with adaptive oxidant defense.
	**Sheppard, 1981b (JMPR, 2003) [34]**	Beagle dogs	Dietary	PQ 0, 7, 20, 60, or 120 mg/kg diet	13 weeks	No treatment-related urinary parameter changes, but distal tubular renal changes were observed at 60 and 120 mg/kg diet; NOAEL = 20 mg/kg diet, equal to 0.55 mg/kg of paraquat ion per kg bw per day in males, and 0.71 mg/kg of paraquat ion per kg bw per day in females.
	**Sotheran et al., 1981 (JMPR, 2003) [34]**	Swiss mice	Dietary	PQ 0, 12.5, 37.5, or 100 mg/kg diet; highest group increased to 125 mg/kg from week 36	Up to 97–99 weeks	Proximal tubular lesions at the highest dietary level included hydropic degeneration, eosinophilia, degeneration, and/or dilatation; mild renal changes were also seen in males at 37.5 mg/kg diet. NOAEL = 12.5 mg/kg diet, equivalent to 1.88 mg of paraquat ion/kg bw per day.
	**Mølck and Friis, 1997 [43]**	Isolated rabbit renal proximal	*Ex vivo*	PQ 0.5 or 5 mM	60–180 min	Paraquat impaired tubular PAH and TEA transport and reduced oxygen consumption,

Pesticide / class	Study	Model / species	Exposure route	Dose / concentration	Duration	Key renal / mechanistic finding
		tubular segments				consistent with mitochondrial dysfunction and secondary transport failure.
	**Wunnapuk et al., 2013 [44]**	Male Wistar rats	Oral gavage	PQ 15, 30, 60, or 90 mg/kg	8, 24, and 48 h (90 mg/kg euthanized at 24 h)	PQ caused proximal and distal tubular necrosis with tubular cell loss, more marked in the medulla; uKIM-1 was the best early predictor of histologic renal injury (AUC 0.81 at 8 h; 0.98 at 24 h), while uCys-C and uAlb also correlated with injury severity.
	**Zheng et al., 2022 [93]**	NRK-52E rat renal tubular epithelial cells	*In vitro*	PQ 300 μM; ISO 5 or 10 μM pretreatment for 30 min	24 h PQ exposure	PQ increased apoptosis and oxidative stress; ISO attenuated injury and upregulated SOX9/TOLLIP signaling.
		Male Wistar rats	IP	PQ 25 mg/kg; ISO 25 or 50 mg/kg pretreatment IP once daily for 7 days	Kidneys assessed 24 h after PQ	PQ induced tubular injury and renal dysfunction; ISO reduced apoptosis, oxidative stress, and SOX9/TOLLIP-associated injury signaling.
	**Hu et al., 2023 [47]**	Human serum; 12 acute PQ-poisoned patients	Clinical poisoning cohort; serum	Not applicable	Single early sampling	Acute PQ poisoning increased PUFAs and oxidized lipid metabolites (HETEs/HODE), consistent with lipid peroxidation and

Pesticide / class	Study	Model / species	Exposure route	Dose / concentration	Duration	Key renal / mechanistic finding
		and 12 healthy controls	sampling within 24 h of exposure			ferroptosis-related injury.
		Human plasma	*Ex vivo* addition to blank plasma	PQ 5 µg/mL ± H_2O_2	48 h	PQ promoted non-enzymatic PUFA oxidation with increased HETEs/HODE formation.
		L-02 cell	*In vitro*	PQ 100 µM	ROS: 2-4h; lipid metabolites/ GSH, Cys, MDA: 12-48h; Fe^{2+}: 12–24 h	PQ increased ROS, Fe^{2+} accumulation, Cys/GSH depletion, mitochondrial injury, GPX4 loss, and ferroptotic cell death.
	**Chen et al., 2024 [48]**	HK-2 cells	*In vitro*	PQ 50, 100, 200, 400, 800, or 1600 µM	36 h	PQ triggered mitochondrial ROS–p38–caspase-1/GSDMD pyroptotic injury, with reduced viability and increased LDH release.
		Male C57BL/6 (WT) mice and GSDMD$^{-/-}$ mice	IP	PQ 50 mg/kg	48 h	PQ increased serum creatinine, BUN, tubular histologic injury, and cleaved GSDMD; global GSDMD knockout and MitoQ attenuated renal dysfunction, oxidative stress, p38 activation, and

Pesticide / class	Study	Model / species	Exposure route	Dose / concentration	Duration	Key renal / mechanistic finding
						pyroptotic signaling.
	**Yu et al., 2025 [49]**	NRK-52E rat proximal tubular cells	*In vitro*	PQ 300 μM; DHC 25, 50, or 100 μM	24 h	DHC attenuated PQ-induced tubular cell injury and supported restoration of autophagic flux, with decreased p62 and increased LC3-II and Atg7; Sox9/Sestrin2 signaling was implicated upstream.
		Male Wistar rats	IP	PQ 25 mg/kg; DHC 2.5, 5, or 10 mg/kg	24 h	DHC reduced PQ-induced renal dysfunction, tubular injury, oxidative stress, and apoptosis, supporting autophagy-mediated protection.
	**Nouri et al., 2025 [94]**	Male Wistar rats	Oral gavage	PQ 25 mg/kg; FA 100 mg/kg	14 days	PQ induced renal dysfunction, oxidative stress, inflammatory signaling, and histologic injury; ferulic acid attenuated these changes.
**Organophosphates**	**Berndt et al., 1984 [67]**	Male Sprague–Dawley rats	SC	DFP 2, 3, or 4 mg/kg	Acute; urine assessed over 0–2.5 h, 2.5–6 h, and 6–24 h after dosing	Transient tubular dysfunction with diuresis, low urine osmolality, natriuresis, glucosuria, and proteinuria; occurred without major renal hemodynamic change or cholinesterase inhibition, supporting a direct tubular transport

Pesticide / class	Study	Model / species	Exposure route	Dose / concentration	Duration	Key renal / mechanistic finding
						effect
	**Kalender et al., 2007 [70]**	Male Wistar rats	Oral gavage	Methyl parathion 0.28 mg/kg bw, single dose	4 and 7 weeks	Renal oxidative injury with increased MDA and progressive glomerular/tubular lesions, including Bowman's space dilatation, glomerular atrophy, tubular dilatation, necrosis, edema, and mononuclear infiltration; vitamin C/E partially attenuated lipid peroxidation.
	**Shah & Iqbal, 2010 [71]**	Male Sprague–Dawley rats	Oral gavage	Diazinon 10, 15, 30 mg/kg/day	8 weeks	Oxidative renal injury characterized by increased lipid peroxidation, depletion of renal antioxidant and glutathione-related defenses, increased BUN/creatinine, and histopathologic damage including glomerular changes and proximal tubular degeneration/necrosis.
	**Baba et al., 2016 [69]**	Wistar rats	CPF by oral gavage; fluoride in drinking water	CPF 1 or 10 mg/kg/day; fluoride 1 or 10 mg/L	28 days	Renal insufficiency with increased BUN/creatinine and oxidative stress in renal tissue (↑MDA, ↓SOD/CAT/GPx), with more pronounced injury during combined fluoride co-exposure, supporting a free-radical-mediated

Pesticide / class	Study	Model / species	Exposure route	Dose / concentration	Duration	Key renal / mechanistic finding
						mechanism.
	**Fuentes-Delgado et al., 2018 [68]**	Male Wistar rats	Oral gavage	Methyl parathion 0.56 mg/kg every third day	8 weeks	Predominant proximal tubular injury with reduced urine flow, glucosuria, phosphaturia, albuminuria, increased urinary γ-GGT, and proximal tubular structural damage (vacuolization, PAS-positive inclusions, brush-border loss), accompanied by altered redox and injury signaling (↓GSH, ↑GPx, ↓TNF-α/BAX).
	**Li et al., 2018 [75]**	HK-2 cells	*In vitro*	Dichlorvos 30 μM	24 h	Proximal tubular cell apoptosis mediated by miR-513a-5p upregulation, Bcl-2 suppression, and activation of mitochondrial apoptotic signaling.
	**Li et al., 2020 [76]**	HK-2 cells	*In vitro*	Malathion 10 μM	48 h	ER-stress-associated tubular apoptosis involving DDIT3/CHOP signaling, modulated by miR-96-5p.
	**Alipanah et al., 2022 [73]**	Male Sprague–Dawley rats	Oral gavage	CPF 3.25, 6.75, or 13.5 mg/kg	15 days	Oxidative-inflammatory injury with ↓ SOD/CAT/GPx/GSH, ↑ MDA, ↑ TNF-α/IL-1β/IL-6, and histopathological lesions in exposed

Pesticide / class	Study	Model / species	Exposure route	Dose / concentration	Duration	Key renal / mechanistic finding
						tissues.
	**Gur & Kandemir, 2023 [74]**	Male Sprague–Dawley rats	Oral gavage	Malathion 100 mg/kg; rutin 50 or 100 mg/kg	28 days	Kidney injury characterized by oxidative stress (↓ SOD/CAT/GPx/GSH, ↑ MDA), ER stress (ATF6, PERK, IRE1, GRP78, CHOP), inflammation (NF-κB, TNF-α, IL-1β), apoptosis (Bax, Apaf-1, caspase-3), and autophagy (Beclin-1, LC3A); rutin mitigated these effects via restoration of antioxidant defense including Nrf2 signaling.
	**Althumairy et al., 2025 [72]**	Male Wistar albino rats	Oral gavage	CPF 10 mg/kg/day; diosmin 50 mg/kg/day	28 days	Tubular-predominant nephrotoxicity with oxidative stress, HMGB1–TLR4/MyD88/NF-κB activation, PPAR-γ/SIRT1 suppression, mitochondrial apoptosis, inflammatory/fibrotic histopathology, and renal dysfunction (↑NGAL, urea, creatinine).
**Atrazine**	**Santa-María et al., 1986 [83]**	Male Wistar rats	Oral gavage	ATZ 100, 200, 400, 600 mg/kg/day	14 days	Urinary Na^+, K^+, and Cl^- excretion increased; creatinine clearance decreased; proteinuria increased, suggesting altered glomerular permeability and/or impaired tubular

Pesticide / class	Study	Model / species	Exposure route	Dose / concentration	Duration	Key renal / mechanistic finding
						reabsorption due to tubular epithelial injury.
	**Jestadi et al., 2014 [81]**	Male Wistar rats	Oral gavage	ATZ 300 μg/kg	15 days	Early renal injury with oxidative stress: ↑ serum creatinine/urea and ↑ renal MDA, with compensatory ↑ SOD/CAT/GPx.
	**Liu et al., 2014 [82]**	Female Wistar rats	Oral gavage	ATZ 0, 5, 25, or 125 mg/kg	28 days	Oxidative stress–mediated tubular injury: at high dose ↑ BUN/creatinine, tubular swelling/hydropic degeneration/vacuolation, ↑ NO/MDA, Nrf2 activation with high-dose failure of HO-1/NQO1/CAT/GSH-Px.
	**Lin et al., 2018 [85]**	Male Kun-Ming mice	Oral gavage	ATZ 50 or 200 mg/kg, with or without lycopene 5 mg/kg	21 days	Oxidative stress–driven tubular injury with mitochondrial damage, AMPK-dependent autophagy activation, and dysregulated p62–Nrf2 signaling; attenuated by lycopene.
	**Wang et al., 2023 [84]**	Female Sprague–Dawley rats	Oral, drinking water	ATZ 0.4, 2, 10 μM in water (approx. 0.013, 0.065, 0.325 mg/kg/day)	6 months	Tubulointerstitial injury/fibrosis without overt functional change: TEC dilatation, tubular atrophy/injury, collagen/α-SMA/vimentin/FN accumulation, ↑ MMP-2/9, Wnt/β-catenin

Pesticide / class	Study	Model / species	Exposure route	Dose / concentration	Duration	Key renal / mechanistic finding
						activation, ↑ TGF-β/TGF-β1/IL-5 and ROS.
	**Rudzki et al., 1989 (JMPR, 2007) [77]**	Sprague–Dawley rats	Dietary	Hydroxyatrazine 0, 10, 100, 300, 600 ppm	13 weeks	Chronic crystal-related nephropathy: ↑ urine volume, ↓ specific gravity, ↑ BUN/creatinine at high dose, with tubular dilatation/atrophy/basophilia, interstitial fibrosis/inflammation, and papillary crystalline casts. NOAEL = 100 ppm, equal to 6.3 and 7.35 mg/kg bw per day in males and female.
	**Chau et al., 1990 (JMPR, 2007) [77]**	Beagle dogs	Dietary	Hydroxyatrazine 0, 15, 150, 1500, 6000 ppm	13 weeks	Chronic crystal-related nephropathy: ↑ urine volume, ↓ specific gravity, ↑ BUN/creatinine at high dose, with tubular dilatation/atrophy/basophilia, interstitial fibrosis/inflammation, and papillary crystalline casts. NOAEL =150 ppm, equal to 5.8 and 6.2 mg/kg bw per day in males and females.
	**Chow & Hart, 1995 (JMPR, 2007) [77]**	Crl:CD(SD)BR rats	Dietary	Hydroxyatrazine 0, 10, 25, 200, 400 ppm	24 months	Persistent crystal-driven renal injury: crystal deposition with tubular/pelvic dilatation, epithelial hyperplasia, papillary fibrosis, chronic

Pesticide / class	Study	Model / species	Exposure route	Dose / concentration	Duration	Key renal / mechanistic finding
						progressive nephropathy, glomerulosclerosis, and mineralization. NOAEL=25 ppm, equal to 0.96 and 1.2 mg/kg bw per day in males and females.
	**Stoker et al., 2013 [87]**	Wistar rats	Dietary	Hydroxyatrazine Males: 11.4, 22.8, 45.75, 91.5, 183.4 mg/kg/day; females: 45.75, 91.5, 183.4 mg/kg/day	Males PND 23–53/54; females PND 22–41/42	Kidney-specific toxicity without endocrine effects: dose-related ↑ kidney weight with hydronephrosis, tubular dilatation, pyelonephritis, and mineralized tubule concretions. LOEL=11.4 mg/kg/day in males, 45.75 mg/kg/day in females.

Abbreviations: GBH, glyphosate-based herbicide; GBHs, glyphosate-based herbicides; bw, body weight; NMDAR, N-methyl-D-aspartate receptor; ROS, reactive oxygen species; NAC, N-acetylcysteine; PND, postnatal day; KIM-1, kidney injury molecule-1; PQ, paraquat; IP, intraperitoneal; NADPH, nicotinamide adenine dinucleotide phosphate; MDA, malondialdehyde; GSH, glutathione; G6PD, glucose-6-phosphate dehydrogenase; JMPR, Joint FAO/WHO Meeting on Pesticide Residues; NOAEL, no-observed-adverse-effect level; PAH, p-aminohippurate; TEA, tetraethylammonium; uKIM-1, urinary kidney injury molecule-1; AUC, area under the curve; uCys-C, urinary cystatin C; uAlb, urinary albumin; ISO, isorhapontigenin; SOX9, SRY-box transcription factor 9; TOLLIP, toll-interacting protein; PUFAs, polyunsaturated fatty acids; HETEs, hydroxyeicosatetraenoic acids; HODE, hydroxyoctadecadienoic acid; GPX4, glutathione peroxidase 4; LDH, lactate dehydrogenase; WT, wild type; GSDMD, gasdermin D; DHC, dehydrocostus lactone; LC3-II, microtubule-associated protein 1 light chain 3-II; SC, subcutaneous; DFP, diisopropylfluorophosphate; CPF, chlorpyrifos; SOD, superoxide dismutase; CAT, catalase; GPx, glutathione peroxidase; BUN, blood urea nitrogen; TNF-α, tumor necrosis factor-α; BAX, BCL2-associated X protein; DDIT3/CHOP, DNA damage-inducible transcript 3/C/EBP homologous protein; NF-κB, nuclear factor kappa B; Nrf2, nuclear factor erythroid 2-related factor 2; HMGB1, high-mobility group box 1; TLR4, toll-like receptor 4; MyD88, myeloid differentiation primary

response 88; PPAR-γ, peroxisome proliferator-activated receptor gamma; SIRT1, sirtuin 1; ATZ, atrazine; HO-1, heme oxygenase-1; NQO1, NAD(P)H quinone dehydrogenase 1; AMPK, AMP-activated protein kinase; α-SMA, alpha-smooth muscle actin; FN, fibronectin; MMP, matrix metalloproteinase; TGF-β, transforming growth factor-β; TEC, tubular epithelial cell; LOEL, lowest-observed-effect level. ↑ indicates increased levels or activity; ↓ indicates decreased levels or activity.
